# Identifying Patterns and Correlates of Unmet Care Needs: The Role of Kinship Networks

**DOI:** 10.1093/geroni/igaf122.2690

**Published:** 2025-12-31

**Authors:** Yao-Chi Shih, Chen-Fen Chen

**Affiliations:** National Taipei University of Nursing and Health Sciences, Taipei, Taipei, Taiwan (Republic of China); National Taipei University of Nursing and Health Sciences, Taipei, Taipei, Taiwan (Republic of China)

## Abstract

Given the aging population and changing family structures, there has been increasing attention on whether adequate support is provided to older individuals in need of long-term care. This study examines how unmet care needs among older persons evolve over time and in what forms. Using data from seven waves of the Taiwan Longitudinal Study on Aging (TLSA), collected between 1996 and 2019, we employed group-based trajectory models to identify developmental patterns of unmet care needs. The study sample was limited to individuals aged 60 and older, focusing on unmet needs related to six activities of daily living (ADLs) and six instrumental activities of daily living (IADLs). Distinct trajectory patterns of unmet needs were identified, including relatively low, gradually increasing, and consistently high unmet needs over time. Structural kinship network did not appear to be as strong as emotional support in explaining unmet needs, controlling for health-related covariates. Despite the apparent need for care and low prevalence of unmet needs, older individuals still reported relatively low demands for additional care. These findings highlight the importance of considering the perspectives of older individuals in understanding unmet care needs. These findings highlight the importance of understanding unmet care needs in the perspective of older people. Potential behavior adjustments and withholding demands for care may carry different policy implications and inform future care strategies.

